# Examination of the diaphragm in obstructive sleep apnea using ultrasound imaging

**DOI:** 10.1007/s11325-021-02472-3

**Published:** 2021-09-03

**Authors:** Viktória Molnár, András Molnár, Zoltán Lakner, Dávid László Tárnoki, Ádám Domonkos Tárnoki, Zsófia Jokkel, Helga Szabó, András Dienes, Emese Angyal, Fruzsina Németh, László Kunos, László Tamás

**Affiliations:** 1 Department of Otolaryngology and Head and Neck Surgery, Semmelweis University, Szigony u. 36, H-1083 Budapest, Hungary; 2 Faculty of Food Science, Szent István University, Budapest, Hungary; 3 Medical Imaging Centre, Semmelweis University, Budapest, Hungary; 4 Institute of Pulmonology, Törökbálint, Hungary

**Keywords:** Obstructive sleep apnea, Ultrasound imaging, Diaphragm, Anthropometrics

## Abstract

**Purpose:**

The aim of this study was to analyze the effect of obstructive sleep apnea (OSA) on the ultrasound (US) features of the diaphragm and to determine if diaphragmatic US may be a useful screening tool for patients with possible OSA.

**Methods:**

Patients complaining of snoring were prospectively enrolled for overnight polygraphy using the ApneaLink Air device. Thickness and motion of the diaphragm during tidal and deep inspiration were measured. Logistic regression was used to assess parameters of the diaphragm associated with OSA.

**Results:**

Of 100 patients, 64 were defined as having OSA. Thicknesses of the left and right hemidiaphragms were significantly different between OSA and control groups. Using a combination of diaphragmatic dimensions, diaphragm dilation, age, sex, and BMI, we developed an algorithm that predicted the presence of OSA with 91% sensitivity and 81% specificity.

**Conclusion:**

A combination of anthropometric measurements, demographic factors, and US imaging may be useful for screening patients for possible OSA. These findings need to be confirmed in larger sample sizes in different clinical settings.

## Introduction

Obstructive sleep apnea (OSA) is the most frequent type of sleep-related breathing disorders, characterized by total or partial collapse of the upper airways during sleep. Severity of OSA can be defined according to the criteria of the American Academy of Sleep Medicine (AASM), based on the apnea–hypopnea index (AHI), which is calculated based on the number of apnea and hypopnea events per hour during sleep [1]. The “gold standard” test for the diagnosis is nocturnal polysomnography (PSG). However, due to its limited accessibility, there is a necessity to find other methods to diagnose patients with OSA.

The diaphragm is the most important skeletal muscle playing a vital role in respiration. It is responsible for 75% of the maximal tidal volume; however, its function and morphology have not been identified in the pathophysiology of OSA [2]. Due to the effort made against the upper airway obstruction, the diaphragm can be fatigued, which may have influence on OSA. However, these effects are different in the several stages of the disorder [3]. The function and contractility of the diaphragm can be investigated using electrodiagnostic testing, although these methods are invasive and they are not easy to carry out in the everyday practice [4]. Ultrasound (US) imaging is not a commonly used diagnostic tool in the assessment of diaphragm dysfunction associated with OSA, even though it is a relatively inexpensive, readily available technique, without the potential risk associated with ionizing radiation. Previous studies have investigated the thickness and motion of the diaphragm in stroke [5], neuromuscular disorders [6], and pulmonary diseases, such as bronchial asthma [7] and chronic obstructive pulmonary disease (COPD) [8], but US characteristics of the diaphragm have not previously been investigated in the diagnosis of OSA. The present study was carried out to analyze the effect of OSA on the US features of the diaphragm and to determine if diaphragmatic US may be a useful screening tool for patients with possible OSA.

## Materials and methods

### Subjects

Of 100 patients (74 men, mean age $\pm$ SD, 42.2 $\pm$ 11.7 years) who visited the Department of Otolaryngology and Head and Neck Surgery of Semmelweis University due to snoring and/or suspected OSA, 64 were defined as having OSA. Only patients over 18 years of age who were complaining of snoring were enrolled. Patients who previously had undergone otorhinolaryngological surgeries, had history of facial trauma or craniofacial deformities (e.g., Down syndrome), had previously diagnosed and/or treated OSA, or were diagnosed as having any systemic muscle or connective tissue diseases, hypo- or hyperthyroidism, pregnancy, systemic neurological or psychiatric diseases, and alcohol or drug abuse, were excluded from this study.

The study was approved by the Hungarian Research Ethics Authority (National Institute of Pharmacy and Nutrition, approval reference number: 2788/2019). All patients gave their written informed consent.

### Sleep test/respiratory pulse oximetry

Overnight polygraphy was performed at the Department of Otolaryngology and Head and Neck Surgery of Semmelweis University, using a Resmed ApneaLink Air device, performed under medical supervision. The results of the polygraphy were analyzed manually, by a sleep specialist, who was blinded to the results of the US examinations. The diagnostic criteria for OSA were defined according to the recommendations of the AASM [1]. Since the present investigation was only aimed to detect OSA, our subjects were classified as controls or patients with OSA.

### Ultrasound imaging

**Fig. 1:**
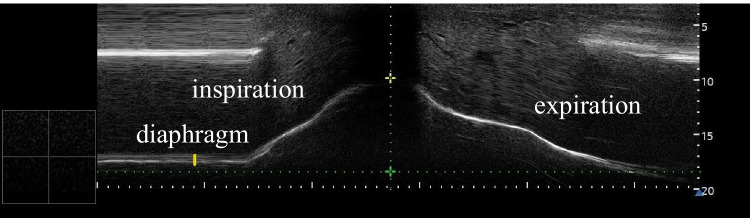
Dilation of the diaphragm, during deep inspiration and expiration, using an M-mode of the US device. (own picture)

The diaphragmatic US examinations were performed by expert radiologists, who were blinded to the results of the polygraphy. The examinations were carried out in a supine position, using a Samsung RS85 device, with L3-12A linear (3-12 MHz) transducers, in a gray-scale B-mode. First, the right hemidiaphragm during tidal breathing was visualized, using a liver window. For this, the transducer was placed under the right ribs, between the central clavicular and axillar lines, and was moved into a dorso-medial and cranial direction, until the posterior parts of the right hemidiaphragm were reached. The cranio-caudal movements of the diaphragm during breathing were assessed, using an M-mode. The left hemidiaphragm was visualized using a spleen window. The transducer was placed subcostally, between the anterior and posterior axillar lines, and the hemidiaphragm was identified as a hypoechogenic structure, located between two hyperechogenic lines. The thickness of the muscle was measured as the distance between its thoracic and abdominal parts. The measurements were repeated three times, during tidal and forced breathing as well, and the parameters were averaged. The maximal displacement of the diaphragm was measured both during forced inspiration and expiration (Fig. 1).

### Statistical analysis

A combination of methods of multivariable statistics [9] and supervised learning [10] increases the efficiency and reliability of diagnostic methods; therefore, in the current study, different multivariable methods, according to Park and Han, were applied [11]. Logistic regression [12] was used to assess the parameters of the diaphragm associated with OSA, using the Blorr R package.

The results of medical imaging are influenced by the subjectivity of the measurements [13], by the biases of them [14] and by the extreme values in the sample [15]. Hence, the application of the traditional statistic methods is limited. Therefore, to rule out biases, robust methods were used for analysis. To create more robust categories, the parameters of the diaphragm were divided into three, equal categories, each of which contained an equal number of patients.

## Results

One hundred patients with snoring and/or suspected OSA were enrolled in this investigation. The most important clinical and statistical features are shown in Table 1.

**Table 1:** Basic statistical data of the two examined groups

Indicators	Range between first and third part of the diaphragm parameters	Control group (*n* = 36)	OSA patients (*n* = 64)	*p* value (based on Bonferroni test)
Gender (male/female)		21/15	53/11	0.000***
BMI [kgm^−2^]		**23.1** ± 3.9	**31.0** ± 2.3	0.000***
Age [years]		**38.1** ± 12.1	**44.4** ± 10.9	0.000***
Diaphragm thickness on right side, at rest [cm]	0.49–0.64	**0.52** ± 0.18	**0.63** ± 0.24	0.020**
Diaphragm dilation on right side, at rest [cm]	1.23–1.75	**1.80** ± 0.97	**1.57** ± 0.61	0.156
Diaphragm dilation during deep breathing, on right side [cm]	6.80–7.99	**7.73** ± 2.17	**7.21** ± 2.26	0.266
Diaphragm thickness on left side, at rest [cm]	0.56–0.70	**0.59** ± 0.15	**0.68** ± 0.20	0.021**
Diaphragm dilation on left side, at rest [cm]	1.40–1.90	**1.82** ± 0.95	**1.90** ± 0.87	0.654
Diaphragm dilation during deep breathing, on left side [cm]	7.50–8.96	**8.27** ± 2.26	**8.13** ± 2.17	0.752

Group A: control group (AHI < 5). Group B: OSA patients (5 ≤ AHI). The parameters are showing the **mean** ± SD values. *** indicates the significant difference at *p* < 0.01, while ** the significant difference at *p* < 0.05

As shown in Table 1, of 100 patients, 64 were diagnosed with OSA, and 36 subjects were investigated as controls. In the OSA group, significantly higher values of the thickness of the right (*p* = 0.02) and the left (*p* = 0.021) hemidiaphragms were measured, contrasted to the parameters of the control group.

The possible effects of age, gender, BMI, and smoking habits on the parameters (i.e., thickness and dilation) of the diaphragm are summarized in Table 2.

**Table 2:** Effects of the different parameters on the values of the diaphragm

Parameters and criteria		**Mean** and SD values	*p* value
Influence of the nutritional status (BMI) on the parameters of the diaphragm (OSA group)			
Diaphragm dilation on right side, at rest [cm]	Normal (A) Overweight (B) Obese (C)	**1.21** ± 0.13 **1.44** ± 0.45 **1.73** ± 0.70	0.074* (A–C)
Influence of the different parameters on the diaphragm (control group)			
Diaphragm dilation on right side, at rest [cm] ^+^	Male Female	**2.14** ± 1.11 **1.33** ± 0.48	0.012**
Diaphragm dilation during deep breathing, on left side [cm]	Male Female	**8.47** ± 2.23 **7.30** ± 2.31	0.04**
Diaphragm thickness on left side, at rest [cm] ^+^	Normal (A) Overweight (B) Obese (C)	**0.57** ± 0.17 **0.55** ± 0.11 **0.70 **± 0.13	0.070* (B-C)
Diaphragm dilation during deep breathing, on left side [cm]	≤ 40 years > 40 years	**8.76** ± 2.18 **7.31** ± 2.17	0.069*
Diaphragm thickness on right side, at rest [cm]	Non-smoker Smoker	**0.49** ± 0.17 **0.68** ± 0.19	0.019**
Diaphragm dilation on right side, at rest [cm]	Non-smoker Smoker	**1.68** ± 0.85 **2.45** ± 1.36	0.078*

The homogeneity of variance assumption was tested using Levene statistic. If there was a difference in variances, Tamhane test was used; if there was not, Bonferroni test was applied. The parameters are showing the **mean** ± SD values. + is showing the significant difference at *p* < 0.05, based on Levene test. * indicates the significant difference at *p* < 0.10, ** is showing the significant difference at *p* < 0.05. Only the significant results are included in the table.

As shown in Table 2, based on the BMI values, the patients were divided into three groups: normal, overweight, and obese patients. Out of the parameters of the diaphragm in patients with OSA, only the dilation of the right hemidiaphragm at rest differed significantly between the normal and obese patients (*p* = 0.074). The other parameters, such as age, gender, and smoking habits, had no significant effect on the parameters of the diaphragm. In the control group, the dilation of the right hemidiaphragm at rest (*p* = 0.01) and the dilation of the left hemidiaphragm during deep aspiration differed (*p* = 0.04), indicating higher values in men. A significant difference was detected in case of the thickness of the left hemidiaphragm, measured at rest between the overweight and obese groups (*p* = 0.07), while the other parameters were not affected by the nutritional status. Between the age groups under and over 40 years, only the dilation during deep inspiration was statistically significantly different (*p* = 0.069). Smoking had considerable effect on the thickness of the right hemidiaphragm (*p* = 0.019), and the dilation measured at rest was also significantly different (*p* = 0.078).

To analyze the parameters for screening patients for possible OSA, logistic regression was used. The limits of the categories are presented in the second column of Table 1. Log-linear analysis was used to analyze the data for categorization of patients with OSA. Input data were transformed into this way and were used for the classification of patients according to OSA/ non-OSA categories by log-linear analysis. The results are presented in Table 3.

**Table 3:** Parameters of the robust classifier algorithm for categorization of patients with OSA, based on the AHI value

Independent variables	Coefficient	Standard error	z	P > z	95% confidence interval (lower range)	95% confidence interval (upper range)
Diaphragm thickness on right side, at rest [category]	1.050	0.474	2.220	0.027^*^	0.121	1.979
Diaphragm dilation on right side, at rest [category]	− 1.555	0.555	− 2.800	0.005^*^	− 2.644	− 0.467
Diaphragm dilation during deep breathing, on right side [category]	− 1.609	0.526	− 3.060	0.002*	− 2.639	− 0.579
Diaphragm thickness on left side, at rest [category]	0.854	0.491	1.740	0.082	− 0.108	1.817
Diaphragm dilation on left side, at rest [category]	0.268	0.417	0.640	0.520	− 0.550	1.086
Diaphragm dilation during deep breathing, on left side [category]	0.158	0.444	0.360	0.722	− 0.713	1.029
Gender	− 2.293	1.181	− 1.940	0.052*	− 4.607	0.022
Age	0.072	0.031	2.350	0.019*	0.012	0.133
BMI	0.227	0.117	1.950	0.052*	− 0.002	0.455
Cons	− 15.444	4.934	− 3.130	0.002	− 25.116	− 5.773

*Indicates the significance level at *p* < 0.05

Using a robust classifier algorithm, a significant outcome was detected in case of the coefficient of the thickness of the right hemidiaphragm (*p* = 0.027) and of the dilation measured at rest (*p* = 0.005) and during deep breathing (*p* = 0.002). In case of the anthropometric parameters, the coefficients of the gender (*p* = 0.052), age (*p* = 0.019), and the BMI (*p* = 0.052) parameters were detected statistically significant.

To select the optimal algorithm and to balance the best model fitting and overfitting, a combination of Akaike information criterion (AIC) and Bayesian information criterion (BIC) by Schwarz were applied. The results of this algorithm are summarized in Table 4.

**Table 4:** Variable selection process for the log-linear model.* AIC* Akaike information criterion, *BIC* Bayesian information criterion. The parameters show mean values

Variable	AIC		BIC	Deviance
BMI	110.423		115.634	106.423
Diaphragm dilation during deep breathing, on right side	105.677		113.493	99.677
Gender	99.592		110.013	91.592
Age	94.511		107.537	84.511
Diaphragm thickness on right side, at rest	92.126		107.757	80.126
Diaphragm dilation on right side, at rest	85.659		103.895	71.659
Diaphragm thickness on left side, at rest	85.335		106.177	69.335
Model performance (# of patients)				
Reference				
			Non-OSA	OSA
Prediction		Non-OSA	29 (81%)	6 (9%)
		OSA	7 (19%)	58 (91%)

As shown in Table 4, AIC and BIC models were contrasted. In case of both algorithms, the prognostic value of the independent variables in OSA, as a dependent variable, was investigated. The aim of this analysis was to find the lowest possible number of parameters, which are sufficient for screening for OSA. The inclusion of the independent variables was based on their role as a risk factor in OSA. The order of the variables was the same in case of the AIC and BIC models as well; therefore, the first variable was BMI, which is an important risk factor for OSA. The dilation of the right hemidiaphragm during deep breathing was selected as the second variable, preceding other basic parameters for screening for OSA, such as age and gender. Based on our algorithm, besides BMI values, the dilation of the diaphragm has an important prognostic value in patients with OSA.

The algorithm applied in this investigation has indicated a normal category in case of 29 subjects (81%) in the control group, while in the other cases (19%), the algorithm showed false-positive outcomes, resulting in categorization of healthy subjects as patients with OSA. These results were based on the anthropometric parameters, e.g., BMI, age, and gender. In the OSA group, 9% of the subjects were defined as false-positive patients with OSA, while 58 patients (91%) were categorized as true-positive cases for OSA. Finally, it can be concluded that the precision of the applied algorithm, based on the BMI, gender, age, and US parameters, can be defined as 87%.

## Discussion

This is the first study which analyzed the effect of OSA on the US features of the diaphragm comprehensively. In our study it was demonstrated that a combination of anthropometric measurements, demographic factors, and US imaging may be useful for screening patients for possible OSA based on robust classifier algorithms.

The motion of the diaphragm has previously been investigated in the literature but there is no comprehensive study which has investigated its role as a radiological marker for OSA. The first study regarding the topic was carried out in 1975. Haber et al. examined healthy subjects and patients suffering from intra-abdominal/subphrenic disorders [16].

Regarding the examination of the diaphragm, only a few previous studies are available. Investigations using other methods (especially electromyography) have been conducted. Luo et al. was able to differentiate between central and obstructive sleep apnea using electromyography and esophageal pressure monitoring [17]. According to the results of Chien et al. by applying electromyography increased fatigue of the diaphragm and of the knee extensors in patients with OSA was found [18]. These examinations offer much information for the diagnosis of OSA, although they are invasive and stressful for the patients.

In our study population, patients with OSA showed significantly higher BMI and age values, contrasted to the control group, which can be explained by the well-known risk factors of the disorder (i.e., older patients with higher BMI are more commonly involved) [19, 20]. The thickness of both hemidiaphragms was significantly higher than in the control group. This phenomenon can be explained by the upper airway obstruction in patients with OSA leading to increased breathing activity. Hence, hypertrophy in the muscles of respiration occurs. Although, based on our investigation, there was no significant difference detected in case of the dilation of the diaphragm between the control and OSA groups, only a tendency for right-sided impaired diaphragmatic dilation, both at rest and during deep breathing, was observed. The possible explanation of this phenomenon might be the limited motion of the thinner diaphragm. The unilateral difference can be caused by the liver.

Boon et al. examined 150 healthy subjects using real-time US imaging. The investigators concluded that the contractility and the thickness of the diaphragm were slightly affected by age, gender, and BMI values of the subjects. The diaphragm was measured during tidal and forced inspiration and expiration [21]. Based on our results, in the OSA group, only the dilation of the right hemidiaphragm at rest differed significantly between normal, overweight, and obese patients. In the control group, only the dilation of the right hemidiaphragm, measured at rest, and the dilation of the left hemidiaphragm during deep breathing were affected by the gender. The nutritional status had only effects on the thickness of the diaphragm. Out of the parameters, the dilation during deep inspiration was affected by the age groups, which confirms the previous findings. However, smoking had a considerable effect on the thickness of the diaphragm and the dilation of the right hemidiaphragm measured at rest, which has not been previously reported. Hida et al. conducted a study in 174 healthy subjects using posteroanterior dynamic chest radiography. According to their results a significant correlation was found between the whole excursion and maximal inspiratory position of the diaphragm although smoking habits and age had no effects on its motion [22]. Yamada et al. concluded that the higher the BMI was measured, the greater the dilation of the diaphragm was detected. In case of older patients, significantly lower dilation of the diaphragm was measured, although other parameters, such as gender and age, showed no considerable effects [23]. Kantarci et al. detected lower motion of the diaphragm using US examination of women, of patients younger than 30 years, and of those with lower BMI parameters [24].

Pazarli et al. conducted a study with 108 patients. Based on the US measurements, it has been concluded that the thickness of the diaphragm was significantly higher in the OSA group contrasting with the results of the control subjects [25] which is similar to our results. However, the authors mentioned above did not investigate the diaphragmatic motion, in contrast to our study. The change in the thickness of the diaphragm (the difference measured at the end of inspiration and expiration) showed a slight positive correlation with the AHI, and the thickening ratio was similar in the OSA and control groups. The main parameters of the study such as the number of involved patients, the age of the subjects, and the distribution of the genders, were similar to the investigation presented by our team.

In order to rule out the effect of subjectivity of the US examination, operator dependency, and measurement errors, a new statistical approach, the so-called robust classifier algorithm, was applied. Using the algorithm a significant outcome was detected in case of the BMI, age, gender parameters, and values of the right hemidiaphragm. Therefore, the prognostic role of these parameters in patients with OSA was verified. Based on both algorithms (AIC and BIC), BMI was detected as the first dependent variable, preceding other parameters, such as gender and age. The preciseness of the algorithm was defined as 87% in the investigated population.

Our results highlight the applicability and relevance of US techniques in the examination of the diaphragm in patients with OSA. It has been concluded that the correlation between the different parameters of the diaphragm and OSA is complex, influenced by different factors. Although using US, a relatively cost-effective non-invasive technique, based on a classification algorithm, the increasing volume of patients with OSA is leading to a necessity to improve the specificity and selectivity of the algorithms contributing to the cost-effective and quick identification of patients with and without OSA.

The diaphragmatic US results may be applied in building algorithms which are suitable for robust and efficient classification of patients. Combination of US and traditional anthropometric methods may present a possibility for screening of patients with OSA.

### Limitations

Our study has limitations. First, due to the relatively low number of patients, the construction of more accurate algorithms is limited. The different degrees of severity of OSA were not taken into consideration.

## Conclusions

A combination of anthropometric measurements, demographic factors, and US imaging may be useful for screening patients for possible OSA. These findings need to be confirmed in larger sample sizes in different clinical settings.
